# Developments in the HCV Screening Technologies Based on the Detection of Antigens and Antibodies

**DOI:** 10.3390/s19194257

**Published:** 2019-09-30

**Authors:** Shrikant Dashrath Warkad, Keum-Soo Song, Dilipkumar Pal, Satish Balasaheb Nimse

**Affiliations:** 1 Institute of Applied Chemistry and Department of Chemistry, Hallym University, Chuncheon 200-702, Korea; shrikant.warkad@hallym.ac.kr (S.D.W.); hanlimsk@empal.com (K.-S.S.); 2 Institute of Pharmaceutical Sciences, Guru Ghasidas Vishwavidyalaya (A Central University), Koni, Bilaspur 495009, India; drdilip71@gmail.com

**Keywords:** anti-HCV antibody, HCV core antigen, NS3, NS4, NS5, HCV detection, viruses, *Flaviviridae*

## Abstract

Hepatitis C virus (HCV) accounts for 15%–20% of cases of acute infection, and chronic HCV infection is developed in about 50%–80% of HCV patients. Unfortunately, due to the lack of proper medical care, difficulty in screening for HCV infection, and lack of awareness resulted in chronic HCV infection in 71 million people on a global scale, and about 399,000 deaths in 2016. It is crucial to recognize that the effective use of antiviral medicines can cure more than 95% of HCV infected people. The Global Health Sector Strategy (GHSS) aim is to reduce the new HCV infections and the HCV associated mortality by 90% and 65%, respectively. Therefore, the methods that are simple, yet powerful enough to detect HCV infections with high sensitivity, specificity, and a shorter window period are crucial to restrain the global burden of HCV healthcare. This article focuses on the technologies used for the detection of HCV in clinical specimens.

## 1. Introduction

**Figure 1:**
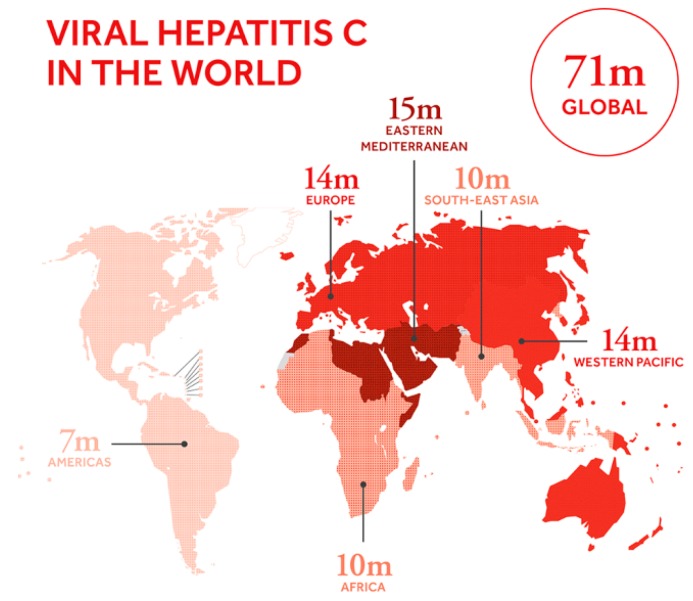
Viral hepatitis C in the world (adopted from the global hepatitis report, 2017. https://www.who.int/hepatitis/news-events/global-hepatitis-report2017-infographic/en/).

Hepatitis C virus (HCV) is an RNA virus of the family *Flaviviridae* that accounts for 15%–20% cases of acute infection, and chronic HCV infection is developed in about 50%–80% of HCV patients. About 15%–30% of patients with chronic HCV infection have a risk of cirrhosis within 20 years. There are 71 million people with chronic hepatitis C virus infection on a global scale, and about 399,000 people died due to hepatitis C in 2016 as shown in Figure 1 [1,2]. Most of the chronically infected patients are asymptomatic and can lead a healthy life. However, the disease develops into fibrosis, cirrhosis, and possibly hepatocellular carcinoma (HCC) in about 20% of chronically infected patients during the period of 10–30 years [3,4,5,6].

The World Health Assembly endorsed the Global Health Sector Strategy (GHSS) on viral hepatitis 2016–2021 in 2016, which targets for the elimination of viral hepatitis as a public threat by 2030. The aim is to reduce the new infections and the associated mortality by 90% and 65%, respectively. The effective use of antiviral medicines can cure more than 95% of HCV infected people, thereby reducing the mortality associated with cirrhosis and liver cancer. However, the limited access to early and accurate screening, diagnosis, and treatment [7,8,9] is the most significant barrier to achieve the goal outlined in the GHSS. A method that allows, rapid, accurate, and highly efficient method for the detection of HCV is crucial for mass screening. Such a technique can prove vital to avoid the transmission of infection and to help physicians to begin the antiviral therapy [10,11].

The most common drawbacks including patient’s anxiety while waiting for test results, adverse effects of treatment and complications related to liver biopsy limit screening for hepatitis C. However, the benefits of HCV screening outweigh these drawbacks. The primary benefit of HCV screening is that the 90%–100% of patients can be treated with current drugs if the infected individuals are identified before the chronic HCV is developed into the advanced fibrosis, cirrhosis, or hepatocellular carcinoma [12,13]. It is vital to notice that the reduction in all-cause mortality is highly associated with the sustained virologic clearance for more than six months after the treatment [14]. Therefore, it is crucial to identify individuals with hepatitis C infection before they develop the symptoms of the disease.

It appears that HCV detection methods are based on molecular assays and serological assays. The molecular assays are RT-PCR based nucleic acid amplification tests (NAT’s) for detection HCV RNA in samples, including blood and other body fluids [15,16,17]. However, the NAT-based assays are not cost-effective, and usually they are not suitable for screening of HCV in a large population for rapid diagnosis [18]. On the contrary, the serological assays are often designed for the detection of HCV antigens, and anti-HCV antibodies in the serum or plasma are quickly taken from venipuncture. Hence, the serological tests are suitable for the mass screening of HCV in the general population. Further, the serological assays are highly applicable to the treatment monitoring and for the confirmation of the virologic clearance [19].

**Figure 2:**
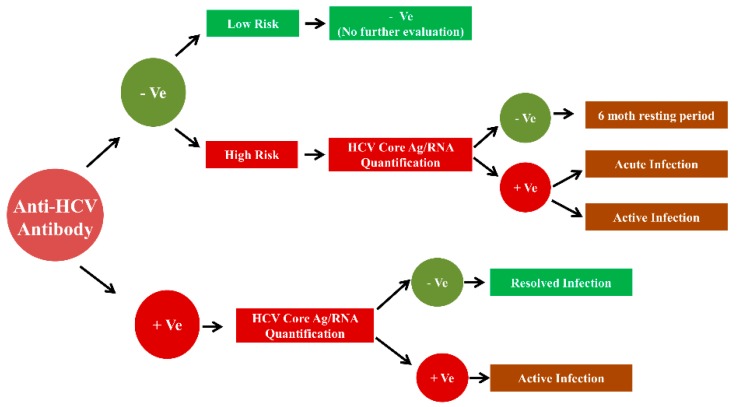
Algorithm for Hepatitis C screening.

In general, the enzyme immunoassays are used to detect the anti-HCV antibodies, antigens in the HCV screening settings. The testing for HCV RNA is then used for the confirmation of HCV infection and identification of specific HCV genotype is standard practice for the diagnostic evaluation of HCV infection, as shown in Figure 2 [20].

**Figure 3:**
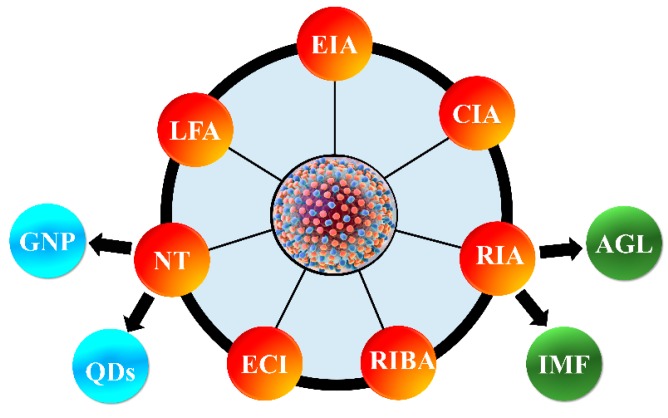
Various methods used for the qualitative and quantitative detection of hepatitis C virus (HCV) antigens, and anti-HCV antibodies for HCV screening. EIA, enzyme immunoassay; CIA, chemiluminescence immunoassay; RIA, rapid immunoassays; AGL, agglutination; IMF, immune-filtration; RIBA, recombinant immunoblot assay; EI, electrochemical immunosensor; NT, nano-metal technology; GNP, gold nanoparticles; QDs, quantum dots; LFA, lateral flow assay.

At present, various serological tests based on enzyme immunoassay (EIA), chemiluminescence immunoassay (CIA), rapid immunoassays (RA) including agglutination (AGL) and immune-filtration (IMF), recombinant immunoblot assay (RIBA), electrochemical immunosensor HCV detection (EI), nano-metal technology (NT) including gold nanoparticles (GNP) and quantum dots (QDs), and lateral flow assay (LFAs) are widely used for the HCV screening in clinical specimens. In many cases, the longer turn-around time, cost, size of the instrument, and necessity of highly trained technicians limits the use of currently available methods in the resource-limited settings [21,22]. Therefore, a low cost, rapid, simple, and accurate method are crucial for sizeable population-based HCV screening. The development of a highly sensitive point-of-care (POC) test for screening of HCV in resource-limited settings is crucial to provide a lower cost of healthcare in developing countries. In this critical review, we have discussed various technologies used for qualitative and quantitative detection of HCV, as shown in Figure 3. This article also elaborates the advantages and disadvantages of the protein-based HCV detection technologies, their efficacy, and future trend to improve their applications in resource-limited settings.

## 2. HCV Proteins and Their Functions

**Figure 4:**
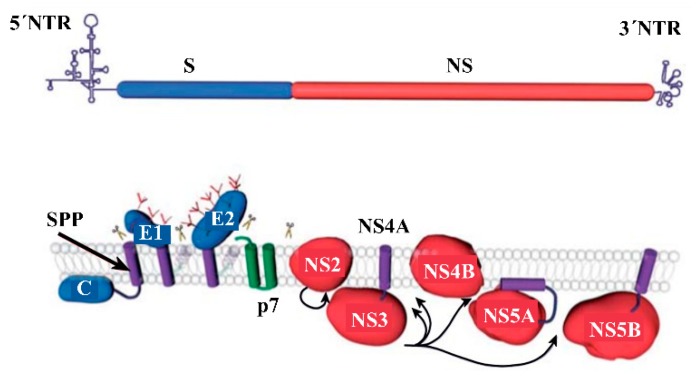
Organization of HCV genome (**top**) and the ten different sections of a polyprotein that are processed to generate HCV specific proteins (**bottom**). Scissors and arrows indicate cleavages by the peptidase to produce ten proteins with unique functions [24].

The genome of HCV encodes a single polyprotein, which is a characteristic of the members of the *Flaviviridae* family [23]. An HCV polyprotein containing 3010 amino acids is processed by cellular and viral proteases to generate ten polypeptides, as shown in Figure 4 [24].

As shown in Figure 4, the HCV genome consists of four regions, including 5’UTR, structural proteins (S), nonstructural proteins (NS), and 3’UTR. A single polyprotein is representing ten different sections for structural proteins C (core), E1, E2, p7, NS2, NS3, NS4A, NS4B, NS5A, and NS5B. The processing of polyprotein by host endoplasmic reticulum (ER) signal peptidase(s) enzymes generates structure proteins. Whereas, the cleavage of polyprotein by HCV proteases generates nonstructural proteins [25]. The information on the structural and non-structural proteins with respect to their genetic stability, function, molar mass, and their applications in HCV screening by detecting them as antigens or their corresponding antibodies are summarized in Table 1.

The core protein is an RNA-binding protein that forms a viral nucleocapsid, and it is cleaved from the C-terminus of polyprotein by a host signal peptidase [34]. The HCV core antigen, a phosphoprotein containing 191 amino acids, is one of the potential diagnostic markers. HCV antibody-based technologies find it challenging to identify the individuals who have resolved their infection and individuals with active disease. However, HCV core antigen tests are designed to detect the circulating HCV core antigen, and thus, these test can diagnose an active infection in patients. Therefore, most HCV screening methods are directed towards the detection of the HCV core antigen [35,36,37,38].

The HCV glycoproteins, E1 and E2, are cleaved from the polyprotein by a host signal peptidase [39]. These glycoproteins are type-I transmembrane proteins with a large N-terminal ectodomain and a C-terminal transmembrane domain [40]. The primary function of E1 and E2 is to bind with the receptor and allowing the entry of HCV into the host cells. Thus these proteins are detected as antigens. The p7 polypeptide is at the junction between the region of the structural and nonstructural protein of the HCV polyprotein, and it acts as an ion channel by localizing on the plasma membrane [41]. The host signal peptidase cleaves the p7 from the HCV polyprotein [39]. The NS2 is an integral membrane protein that is not essential for the formation of the replication complex [42,43]. However, from the structure of NS2, it is revealed that it is a dimeric cysteine protease with two active sites [44]. The NS3 is co-expressed with NS4A, and it is found in association with ER or ER-like membranes. When NS3 is expressed alone, it is distributed in the cytoplasm and nucleus [45]. Further, it is found that the NS3 is a bifunctional protein with serine protease and RNA helicase activity. The NS4A acts as a protease cofactor and contributes one beta-strand to the N-terminal protease domain [46].

The NS4B is a highly hydrophobic nonstructural protein, possibly containing four transmembrane regions. The N-terminal and C-terminal of NS4B are known to be localized in the cytosol. Whereas, a fraction of the N-terminal is also being found in the ER lumen [61,62]. NS4B functions as a component of the viral replicase complex. The NS5A functions as a cofactor for NS5B, and it is found to regulate the response to INF-α treatment. The NS5A is a membrane-associated protein that contains an α-helical structure at its N-terminal, which serves as an anchor to attach on the cell membranes. Similar to most of the HCV proteins, the NS5A is detected in association with ER or ER-derived membranes [63,64]. NS5B is a membrane-associated protein. However, in contrast with the NS5A, the transmembrane region of the NS5B is on the C-terminal [65], and it plays a vital role during the RNA replication in cell cultures [66]. Analogous to NS5A and most HCV proteins, the NS5B is also detected in association with ER or ER-derived membranes [67].

Shown in Table 2 are various technologies that are used for the detection of HCV antigens and anti-HCV antibodies in clinical specimens. The advantages and disadvantages of these technologies are discussed in the following sections.

## 3. Enzyme Immunoassay (EIA)

**Figure 5:**
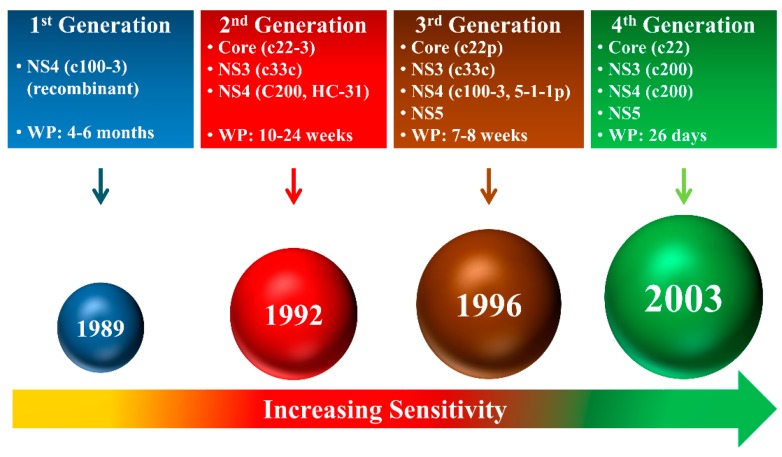
Generations of the protein-based HCV assays (WP, window period).

As of now, the assays for the detection of the HCV antigen and anti-HCV antibody have evolved through four generations of HCV immunoassays to identify HCV infected patients as shown in Figure 5 [7].

Since 1989 with the beginning of first-generation immunoassays, wherein the recombinant c100-3 epitope from the NS4 region was used for the identification of HCV infected patients. However, the window period (WP) from the incident infection to the detection of the protein was 4–6 months. The first-generation assays lacked the sensitivity and the specificity. The second generation assays were developed to overcome the drawbacks of the first generation assays. The second generation assays developed in 1992 incorporated epitopes c22-3, c33c, c200, and HC-31 from the HCV core, NS3, NS4, and NS4 regions, respectively. The window period was successfully reduced to 10–24 weeks. The sensitivity of second-generation assays for HCV detection was significantly improved, making them applicable in clinically relevant settings [7,68,69,70]. The third-generation assays were developed in 1996 with a basic principle of detection of anti-HCV antibodies in plasma or serum against several HCV protein epitopes. The third-generation assays were multi-target format and included detection of antigens from the core (c22p), NS3 (c33c), NS4 (c100-3, 5-1-1p), and NS5 regions. The multi-target based assays demonstrated enhanced performance than the previous generations and were more effective with the ability to reduce the window period to 7–8 weeks [7,68,71,72]. However, the drawback of the assays in this generation was that the low positive predictive values in a low prevalence of HCV infection (<10%) [73,74].

**Figure 6:**
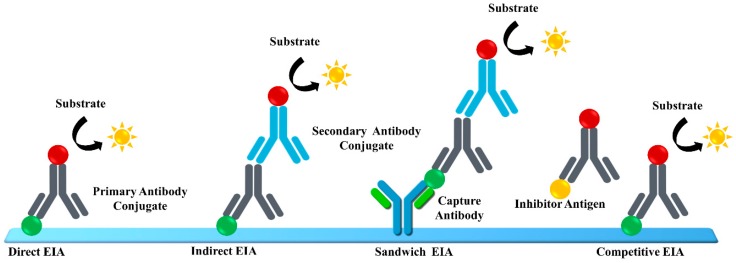
Methods for the detection of anti-HCV antibodies using enzyme immunoassays.

The fourth-generation assays, more commonly known as the antigen–antibody combo assay, simultaneously detect the HCV antigen and antibody. These assays are more convenient as two HCV markers are identified in the same test. The fourth-generation assays provide a single platform for the detection of antigen and antibodies in human sera. Hence, they are highly applicable in resource-constrained settings. Further, it is essential to notice that the fourth-generation assays are very sensitive as the window period is reduced to an all-time low of 26 days. Recently, a fourth-generation assay was reported to increase reactivity for the detection of antigens derived from the core, NS3, NS4A, NS4B, and NS5A regions for the detection of HCV genotypes 1a and 1b. Moreover, the improved detection of NS3 and NS4 antigens allows highly sensitive detection of HCV genotypes 2 and 3a [28]. There are several reports on the detection of anti-HCV antibodies using enzyme immunoassays for hepatitis C testing, as shown in Figure 6 [75,76,77,78].

The HCV immunoassays can be divided into three categories, those targeting anti-HCV antibody detection and those targeting the HCV core antigen detection, and those targeting multiple HCV antigen detection.

### 3.1. Anti-HCV Antibody Detection

Lopes et al. reported the evaluation of an EIA for anti-HCV antibody detection using single antigen [79]. A recombinant c22 antigen was localized on the solid-phase and allowed to complex with anti-HCV antibodies in serum samples to detect anti-HCV IgG. The complexes were detected by using horseradish peroxidase goat anti-human IgG. This method demonstrated sensitivity and specificity of 95% and 97%, respectively, in a study of 145 healthy controls and 106 patients with confirmed HCV infection.

Tests for anti-HCV antibodies are usually qualitative with either a positive or a negative result. However, several studies have found that the specimen with low signal/cutoff (S/C) ratios commonly assigned false-positive results. Dufour et al. investigated specimens with low positive results (S/C ratios ≤ 3.7) with recombinant immunoblot assay and found that 86% of the samples were HCV negative. Therefore, they recommended that the laboratories should report the S/C ratio for anti-HCV EIA results. Moreover, supplemental RIBA testing should be performed for the specimens with low-positive values to avoid reporting false-positive results [48].

### 3.2. Core Antigen Detection

An immunoassay that detects and quantifies the total HCV core protein in serum was reported to detect the core antigen in the antibody-negative early phase of hepatitis C infection specimens. Hence, this assay is highly applicable for HCV screening blood banks for the screening of blood products [80,81]. Massaguer et al. reported on the application and the performance of HCV core antigen immunoassay for the monitoring of a viral load after liver transplantation [82]. It is known that the measurement of HCV-RNA concentration provides crucial information on viral load in the liver transplantation settings. However, besides being expensive, HCV-RNA testing is not routinely available in all laboratories. Massaguer et al. found that the quantification of HCV core antigen is appropriate for monitoring the viral load in HCV-infected patients undergoing liver transplantation. Alzahrani et al. also reported that the screening of blood donors by HCV core antigen assays has high potential in minimizing the risk of using HCV positive blood from a patient with the early phase of hepatitis C infection [83].

### 3.3. Multiple Antigen Detection

The current standard in diagnosing HCV infection is a two-step approach that requires screening with an anti-HCV antibody test followed by HCV-RNA detection to confirm the infection [84]. One of the reasons behind this two-step approach was the low specificity and sensitivity of currently available HCV core antigen assays [85,86]. Therefore, to improve the sensitivity and specificity of the HCV antigen assays so that they can be applied for the one stem detection of HCV infection, Hu et al. proposed the use of a test that simultaneously detects HCV core antigen and HCV nonstructural proteins [87,88]. The assay for the detection of multiple HCV antigens showed 98.9% specificity and 100% sensitivity compared to serum anti-HCV antibody assay and HCV-RNA detection assays.

## 4. Chemiluminescence Immunoassay (CIA)

The CIA uses a luminescent molecule, which serves as an indicator of the analytic reaction by emitting the visible or near-visible (λ = 300–800 nm) radiation. In many cases the enzymes used in CIA convert a substrate to a reaction product that emits photons. The CIA test is an epitope-specific antibody detection test and generally shows a very similar sensitivity and specificity to the third-generation EIA test [89]. 

**Figure 7:**
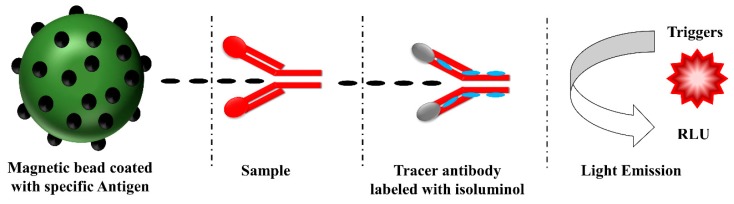
CIA principle in the diagnostic testing of autoantibodies [90].

A principle behind the diagnostic testing of antibodies by using CIA is depicted in Figure 7. The antigens specific to the anti-HCV antibodies are coated on the magnetic beads. The addition of samples to the solution containing magnetic bead coated with antigens allows capturing the anti-HCV antibodies. The captured anti-HCV antibodies are then detected with the tracer antibody labeled with isoluminol. Upon enzymatic reactions catalyzed by peroxidase, the substrate is converted into a reaction product that emits photons, and then the emitted photons are detected by using a detector [90]. The CIA method is successfully applied for the screening of HCV infected patients by detecting anti-HCV antibodies. The automated CIA assays show high precision, high reliability, short turn-around time, and are technically simple due to full automation [89].

### 4.1. Anti-Hcv Antibody Detection

Currently, various commercial automatic CIA assays are available for the detection of anti-HCV antibodies in clinical laboratories [49]. These assays are replacing the conventional EIAs, particularly in high-volume clinical laboratories because of the automation and high sensitivities. The CLA exhibited considerably increased sensitivity and specificity, high positive predictive value compared to those of EIA for the detection of anti-HCV antibodies [91]. Various commercial assays available for the detection of the anti-HCV antibodies based on the CIA method are listed in Table 3.

Kim et al. evaluated the performance comparison of four anti-HCV CIAs, including the Architect Anti-HCV assay, the Vitros Anti-HCV assay, Access HCV Ab PLUS assay, and the Elecsys Anti-HCV assay. According to their report, these assays showed good agreement for anti-HCV antibody detection with a range of 94.5% to 98.1%. The specificities of 98.8%, 96.5%, 98.2%, and 98.2% were found for Architect, Vitros, Access, and Elecsys assays, respectively [92]. Ismail et al. evaluated the performance of fully automated, enhanced CIA assay for the detection of anti-HCV antibody and found the sensitivity and specificity of 98.9% and 97.2%, respectively [91]. Tang et al. reported a systematic review and meta-analysis to evaluate the diagnostic accuracies of EIA based assays [93]. Feng et al. assessed the HISCL Anti-HCV assay, which is based on CIA technology and found that this assay showed 98.97% and 100% sensitivity and specificity, respectively, for the detection of HCV infections in clinical samples [94].

### 4.2. Core Antigen Detection

**Figure 8:**
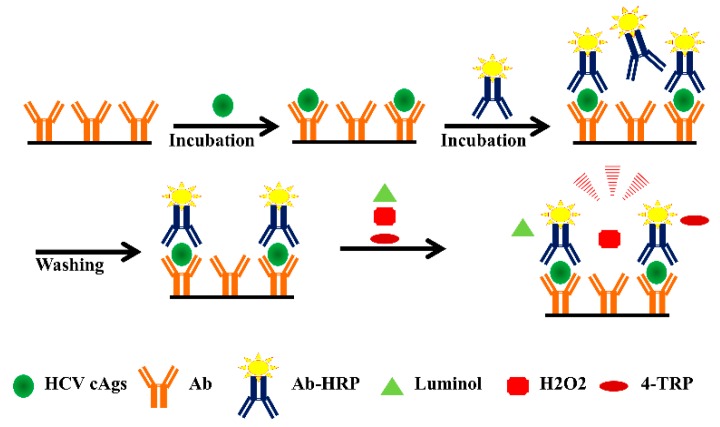
Schematic illustration of a CIA used for the detection of the HCV core antigen.

It is crucial to notice that the HCV core antigens can be detected in clinical samples during the window period with an automated CIA. Therefore, the development of highly sensitive CIA methods for the detection of HCV antigens is crucial for the identification of the HCV in the early stage of infection. A typical CIA based method used for the detection of HCV core antigen is depicted in Figure 8.

There are limited research reports on the use of CIA for the detection of HCV antigens in order to identify the people with HCV infections. Muerhoff et al. detected HCV core antigen in human serum and plasma with an automated CIA and showed a 99.9% specificity and sensitivity [35]. Morota et al. reported the microparticle CIA for the quantitative determination of HCV core antigen and found the specificity of 99.8% by testing 5403 specimens [50]. Rockstroh et al. reported the results of HCV core antigen CIA assay in comparison with the HCV RNA test. They found the concordance of 99.5% and 99.24% between the HCV core antigen and HCV RNA in pre-treatment samples and post-treatment week 12 samples. The specificity in anti-HCV positive HCV RNA negative samples tested was 100% [95]. Liu et al. used the p-phenol derivative, 4-(1,2,4-triazol-1-yl)phenol (4-TRP) as an efficient enhancer of the luminol–hydrogen peroxide (H2O2)–horseradish peroxidase (HRP) in the CIA system for detection of the HCV core antigen. Their method showed a good linear relationship for the HCV core antigen concentration in the range of 0.6–3.6 pg/mL [96]. There are no reports on the detection of multiple HCV antigens using the CIA method.

## 5. Rapid Immunoassays (RIA)

Rapid immunoassays are defined as those that have the following characteristics, (i) turn-around time till the result is less than 30 minutes, (ii) moderately complex assays that do not need to follow the Clinical Laboratory Improvement Amendments of 1988 (CLIA’88), and (iii) an assay that does not require specialized equipment [97,98]. The majority of rapid immunoassays (RIA) assays are applicable in point-of-care settings, and they are manual single-use devices. The RIA designs that are used these days are latex agglutination, immunofiltration (flow-through), immunochromatographic (lateral flow), and optical immunoassays (OIA) [99]. The agglutination and immunofiltration assays will be briefly introduced in this article, and the lateral flow assays will be described in a separate section. The RIA tests are developed for the detection of anti-HCV antibodies associated with core, NS3, NS4, and NS5 regions as well as antigens of the virus [98]. The RIA is suitable where the infrastructure and the laboratory expertise are limited [100].

The agglutination (AGL) based method uses particles that are coated with an analyte-specific capture antibody. The analyte in a test sample triggers the formation of aggregates that can be visible to the naked eye. The lack of sensitivity and specificity are the major drawbacks of the agglutination-based assays. However, due to their rapid nature, low cost, and requirement of minimal reagents, they are used widely [101]. The agglutination based RIA are reported for the detection of antibodies only.

Immunofiltration (IMF) assays, commonly known as flow-through immunoassays, developed for the detection of anti-HCV antibodies. The antigens specific to the anti-HCV antibody are immobilized on the porous immunofiltration membrane. When a sample is applied to the membrane, it passes through the membrane, and the anti-HCV antibodies in the samples bind to the immobilized antigens. The captured antibodies are then detected with the anti-HCV antibody specific immunoglobulin G that are designed to produce distinct colors on the region.

### 5.1. Anti-HCV Antibody Detection

Daniel et al. reported the RIA that was intended for the detection of anti-HCV antibodies. In comparison to EIA, RIA demonstrated sensitivity and specificity of 99.3% and 99.0%, respectively [102]. Firdaus et al. evaluated the performance of RIA in clinical samples. Around 15.74% of these samples were HCV seropositive by ELISA, and 11.02% were RNA positive by nested RT-PCR. Therefore, the results of their study showed that the RIA alone could not be relied on as an absolute diagnostic tool for screening HCV [103]. 

### 5.2. Core Antigen Detection

Mikawa et al. reported a rapid one-step immunochromatographic assay for the detection of HCV core antigen detection. For this method, they expressed in the HCV core antigen in *Escherichia coli* a recombinant fusion protein with glutathione S-transferase (GST). The expression of a correct protein was confirmed by immunological detection with HCV positive serum. This method was capable of detecting 0.25–12.0 μg of the recombinant protein [57].

## 6. Recombinant Immunoblot Assay (RIBA)

The recombinant immunoblot assay can detect the anti-HCV antibodies present in the blood of HCV infected patients. RIBA was mainly used as a secondary confirmation test when the first-line screening test for HCV showed positive or indeterminate results. However, the use of RIBA based assays was discontinued because of the improved sensitivities of other methods used for HCV detection. However, before its discontinuation, RIBA detected anti-HCV antibodies using the recombinant antigens and synthetic peptides from a core, NS3, and NS5 proteins for immobilization onto a membrane [104,105]. Due to its robust specificity, this method was used as additional serological testing, and it became clinically obsolete with the availability of molecular tests [106]. Even though there are several reports on the application of RIBA for the detection of HCV infected patients, other immunoassays with higher sensitivity and specificity took precedence [107,108,109,110].

## 7. Electrochemical Immunosensors (EI)

Similar to any other immunoassay, the sensitivity and specificity of EI based assays depend on the highly specific molecular recognition between antigens and antibodies. The EI based assays have attracted massive attention from the scientific community for their fast and highly sensitive detection antigens [111,112]. The advantages of EI are high sensitivity, short turn-around time, and cost-effectiveness [113,114].

**Figure 9:**
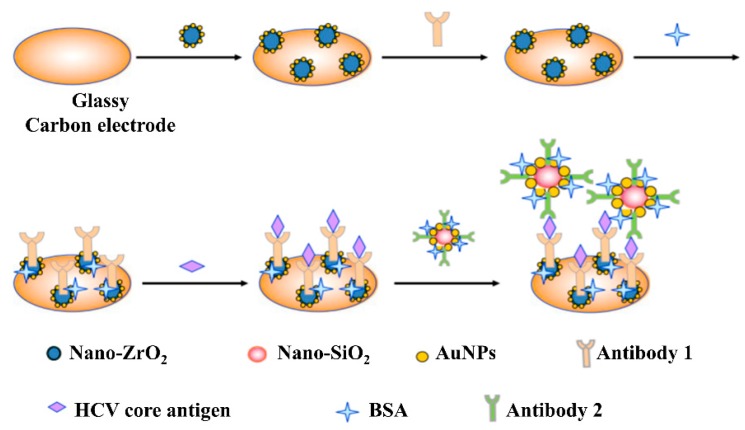
Schematic illustration of the electrochemical immunosensor construction process and its application in the detection of HCV core antigen [115].

Figure 9 depicts a typical fabrication method of an EI and its application in the detection of an analyte. As shown in Figure 9, the nanocomposites of AuNPs/ZrO_2_-Chits and AuNPs/SiO_2_-Chits were used for the detection of the HCV core antigen. The primary HCV core antibodies are immobilized on the AuNPs/ZrO_2_-Chits nanocomposite modified on glassy carbon electrode (GCE). Whereas, the secondary antibodies are immobilized on the AuNPs/SiO_2_-Chits nanocomposite. Cyclic voltammetry is used to detect the formation of the sandwich-type complex upon addition of the sample. The EI based assays are known to exhibit high sensitivity, selectivity, and good reproducibility [115].

### 7.1. Anti-HCV Antibody Detection

Zhao et al. recently reported a portable EI assay for the multiplexed detection of antibodies against the HIV core antigen and HCV core antigen in serum [116]. The EI assay used microfluidic paper-based electrochemical immunosensor for detection of antibodies in eight samples at a time within 20 min. The portable, low-cost, easy to use, and high-throughput are the few advantages of this method.

### 7.2. Core Antigen Detection

Ma et al. reported an ultrasensitive and selective EI for the detection of the HCV core antigen. For the development of EI, they used graphitized mesoporous carbon–methylene blue (GMCs–MB) nanocomposite as an electrode. The horseradish peroxidase-DNA-coated carboxyl multi-wall carbon nanotubes (CMWNTs) were used as a secondary antibody layer. Under optimum conditions, the EI exhibited a detection limit of 0.01 pg mL^−1^ with high selectivity [117]. Valipour et al. reported a label-free EI for ultrasensitive detection of HCV core antigen in serum samples. With the help of a modified glassy carbon electrode, the linear detection range of 0.08–110 pg mL^−1^ with the detection limit of 10 fg mL^−1^ was achieved [118].

### 7.3. Detection of Other Antigens

Liang et al. developed a sandwich immunoassay for the detection of HCV NS5a protein using a glassy carbon electrode modified with an au-moo3/chitosan nanocomposite. The assay showed a wide detection range of 1 to 50 µg mL^−1^ with the detection limit of 1 ng mL^−1^ [119].

## 8. Nanotechnology

The remarkable progress in nanoscience and nanotechnology in the last decade has opened a new way of developing assays for the identification of HCV infection with high sensitivity and specificity [120]. The use of nanomaterials and nanoparticles such as gold nanoparticles (GNP), quantum dots (QDs), silver nanoparticles, carbon or silica nanoparticles, and magnetic beads had led the way for the development of highly sensitive immunosensors [121,122,123,124,125]. In this article, only the methods that use GNP or QDs for the detection of HCV were discussed.

### 8.1. Anti-HCV Antibody Detection

**Figure 10:**
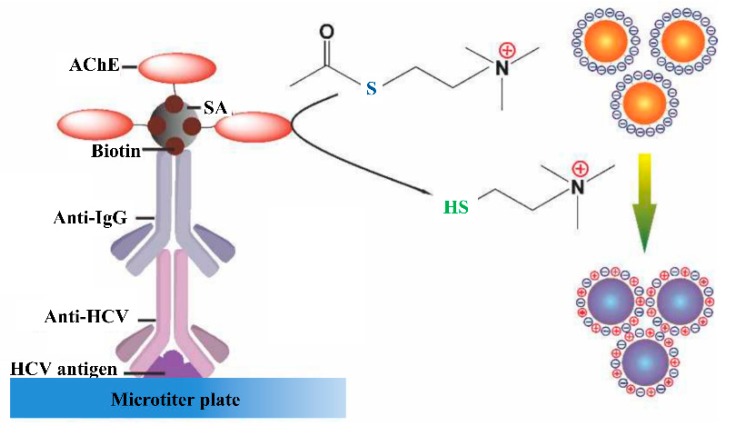
The GNPs in the solution phase can be triggered to form aggregates via an electrostatic interaction by thiocholine generated from AchE catalyzed hydrolysis reaction in the presence of anti-HCV antibody (adopted from Cheng Y, Tang H, Jiang J. Anal. Methods, 2017, 9, 3777–3781).

Recently, Cheng et al. developed a technique that allows the enzyme-mediated assembly of GNPs for the colorimetric detection of the anti-HCV antibody, as shown in Figure 10 [126].

As shown in Figure 10, the aggregation of GNPs induced by the acetylcholinesterase-catalyzed reaction allows colorimetric detection of the anti-HCV antibody with the detection limit of 10−13 g mL^−1^ anti-HCV antibody. Duan et al. developed an assay for rapid and simultaneous detection of anti-HBV and anti-HCV antibodies on a protein chip using nano-gold immunological amplification and the silver staining method. The assay used a mixture of NS3, NS5, and HCV core antigens for the detection of anti-HCV antibodies with the detection limit of 3 ng mL^−1^ [53]. Liu et al. reported the protein array-based detection of HCV using QDs. The anti-HCV antibody in serum was detected by immobilizing the highly purified HCV NS3, NS4, NS5, and core antigens on the surface of encoded beads. A clinical study using this method found that the sensitivity, specificity, and accuracy were 97.5%, 96.0%, and 97.1%, respectively [127].

### 8.2. Core Antigen Detection

Yin et al. developed an assay for HV core antigen detection based on the GNP probes. In this method, anti-HCVcAg monoclonal antibodies were functionalized on the magnetic microparticles probes were with that recognize and bind HCV core antigen. The GNPs were modified with the polyclonal antibody and the barcode single-stranded DNA (ssDNA). The HCV core antigen in the samples allows the formation of GNP-HCV core antigen-MMP sandwich immuno-complex, which is then separated magnetically. The magnetically separated immuno-complex with the barcode ssDNA is characterized by real-time PCR for the quantification of HCV core antigen. The detection limit of this method was reported to be 1 fg mL^−1^ [54].

### 8.3. Detection of Other Antigens 

Roh et al. proposed the QDs-supported RNA oligonucleotide method for the detection of HCV NS5B protein using a biochip with high sensitivity and specificity. Their approach allowed to detect the HCV NS5B viral protein in the range of 1 µg mL^−1^ to 1 ng mL^−1^ and a limit of detection of 1 ng mL^−1^ [56,128]. Roh et al. reported a technique that uses a nanoparticle-supported aptamer probe. The target HCV NS3 was detected visually by using the QDs based RNA aptamer with the detection limit of the 5 ng mL^−1^ level [55].

## 9. Lateral Flow Assay (LFA)

**Figure 11:**
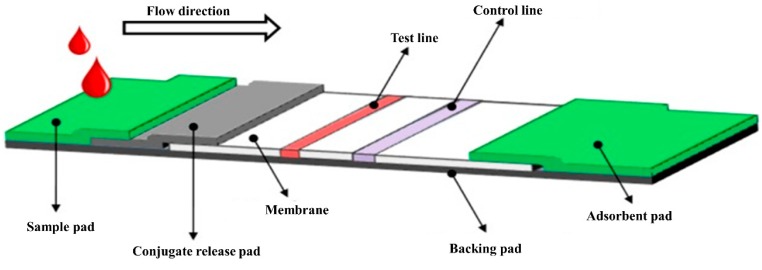
A typical structure of a lateral flow immunoassay test strip [129].

Lateral flow assay, also known as immuno-chromatographic assays (ICA), is gaining high interest recently because they are low-cost, simple, rapid, and allow the integration with portable detection devices. The LFAs are performed on a membrane strip with various parts including the application pad, conjugate pad, nitrocellulose membrane, and adsorption pad assembled on a plastic backing [129]. In general, the nitrocellulose membrane contains two lines, one each for an analyte and internal control, as shown in Figure 11.

### 9.1. Anti-HCV Antibody Detection

Xiang et al. reported a double antibody sandwich-lateral flow immunoassay for the rapid and straightforward detection of HCV. They screened several recombinant proteins, including HCV core antigen, E1, E2, P7, NS2, NS3, NS4A, NS4B, NS5A, and NS5B for the highly specific screening of HCV. In this study, they found that the full-length core and NS3 proteins have the dominant immunodominant epitopes of the HCV genome that were used for the development of LFA [58]. Kosack et al. evaluated the diagnostics accuracy of the ImmunoFlow HCV from Core Diagnostics for the detection of anti-HCV antibodies. The assay demonstrated 100% sensitivity and 100% specificity [130]. There are several reports on the OraQuick HCV rapid antibody test. Lee et Gao et al. reported the overall specificities of OraQuick HCV test to 99.6%–99.9% [131]. Cha et al. also evaluated the OraQuick HCV test for its performance in the detection of HCV and found the clinical sensitivity and specificity of 97.8% and 100%, respectively [132]. The sensitivity and specificity of this method were 94.1% and 99.5%, respectively [133].

### 9.2. Core Antigen Detection

Wang et al. developed the method for detection of the HCV core antigen by using a highly specific aptamer. The detection limits for their method were found to be 10 pg mL^−1^ and 100 pg mL^−1^ by using scanner detection and detection with naked eyes [134].

## 10. Conclusions

The development of technologies for the identification of HCV infected patients has been a hot topic of research for the last three decades. It is crucial to screen the blood donors for HCV infection to avoid the spread of HCV. Furthermore, it is crucial to screen organ donors in liver transplant surgeries. Beyond this, the screening of HCV in the general population is of paramount importance to control hepatitis associated illness including fibrosis, cirrhosis, and possibly hepatocellular carcinoma, because they are preventable. In many cases, it is crucial to detect HCV infection during the window period. It is evident from the presented articles that research on the HCV detection methods has reduced the window period from six months to 26 days. Therefore, the current study should be focused on the ultrafast HCV screening methods that not only demonstrate high sensitivity and specificity but also they should demonstrate the ability to detect the HCV as early as in less than a week after infection. It is known that the effective use of antiviral medicines can cure more than 95% of HCV infected people. Nonetheless, only screening of HCV is not enough for combating the HCV infection because, for the successful treatment of hepatitis, it is important to know the HCV genotype. Therefore, future efforts should be engaged towards the simplistic yet highly specific and sensitive methods that not only screen for HCV but also allow the HCV genotyping simultaneously.

## Figures and Tables

**Table 1:** HCV protein, their functions, molar mass, and their applications in HCV screening by detecting them as antigens or their corresponding antibodies.

HCV Proteins	Genetic Stability	Function	Molar Mass	HCV Antigen Detection?	Anti-HCV Antibody Detection?
**Structural Proteins**					
Core (C): P22	Stable	A significant component of viral nucleocapsid, Binds viral RNA during assembly	20 KDa [26]	Yes [27]	Yes [28]
E: gp 35 envelope glycoproteins	A high degree of genetic diversity	Receptor binding and HCV entry into target cells	31 kDa [26]	Yes [27]	-
E2: gp 70 envelope glycoproteins			62 kDa [26]	Yes [29,30]	-
**Non-Structural Proteins**					
NS1: p7 small polypeptide	Stable	Ion channel localized to plasma membrane	7 KDa [24]	-	-
NS2: p23	-	Component of NS2-3 proteinase	21 KDa [24]	-	-
NS3: p70	-	Serine protease and RNA helicase	69 KDa [24]	Yes [31]	Yes [7]
NS4A: p8	Stable	Protease cofactor	6 KDa [24]	-	Yes [32]
NS4B: p27	Stable	Proteins	27 KDa [24]	-	Yes [7]
		Components of the viral replicase complex			
NS5A:p56/58	Stable	Cofactor for NS5B	56 KDa [24]	Yes [33]	Yes [7]
		Regulate response to INF-α treatment			
NS5B: p68	Stable	RNA dependent polymerase	68 KDa [24]	-	Yes [7]

**Table 2:** The technologies used for the detection of HCV proteins either as HCV antigen or the anti-HCV antibodies for the HCV screening.

Technology	HCV Proteins	Core	E1	E2	NS1	NS2	NS3	NS4A	NS4	NS5A	NS5B	Reference
EIA	Ab	✓					✓	✓	✓	✓	✓	[31,47,48]
	Ag	✓					✓					
CIA	Ab	✓					✓	✓	✓	✓		[28,35,49,50]
	Ag	✓										
RA	Ab	✓					✓	✓		✓		[21,51]
	Ag											
RIBA	Ab	✓		✓			✓	✓	✓	✓	✓	[30,52]
	Ag											
EI	Ab											[52]
	Ag	✓								✓		
NT	Ab	✓					✓			✓		[53,54,55,56]
	Ag	✓					✓			✓		
LFA	Ab	✓					✓			✓		[57,58]
	Ag	✓										
ABA	Ab											[29,59,60]
	Ag	✓		✓								

Ag: HCV-antigen; Ab: Anti-HCV antibody.

**Table 3:** Characteristics of automated anti-HCV antibody assays approved for in vitro diagnostics.

Assay	Assay Principle	Solid Phase	HCV Antigen	Reaction Time (min)
Architect Anti-HCV, Abbott Laboratories	ECIA	Paramagnetic Particles	Core, NS3, NS4	29
The LIAISON® XL murex HCV Ab, DiaSorin	CIA	Paramagnetic Particles	Core, NS3, NS4	46
Vitros Anti-HCV, Ortho Clinical Diagnostics	CIA	Microwell	Core, NS3, NS4, NS5	55
Elecsys Anti-HCV, Roche Diagnostics	ECA	Paramagnetic Particles	Core, NS3, NS4	18
ADVIA Centaur HCV Assay, Siemens	CIA	Magnetic Particles	C22-3 (core), NS3, c200, NS5	58
Access HCV Ab PLUS, Bio-Rad Laboratories	CIA	Paramagnetic Particles	Core, NS3, NS4, NS5	55
